# Pediatric Abdominal Tuberculosis: A Multicenter Experience from an Endemic Region

**DOI:** 10.12669/pjms.42.(11AASC).15795

**Published:** 2026-04

**Authors:** Javeria Javed, Muhammad Osama Khan, Fatima Zahra, Rashida Abbas Fakhruddin Lacewala, Maryam Aftab, Samira Imran, Humza Thobani, Muhammad Aqil Soomro, Saqib Qazi, Saleem Islam

**Affiliations:** 1 Dr. Javeria Javed, MBBS. Section of Pediatric Surgery, Department of Surgery, Aga Khan University, Karachi, Pakistan; 2 Dr. Muhammad Osama Khan, MBBS. Section of Pediatric Surgery, Department of Surgery, Aga Khan University, Karachi, Pakistan; 3 Fatima Zahra, Medical College, Aga Khan University, Karachi, Pakistan; 4 Rashida Abbas Fakhruddin Lacewala, Medical College, Aga Khan University, Karachi, Pakistan; 5 Dr. Maryam Aftab, MBBS, FCPS. Indus Hospital and Health Network, Indus Hospital, Karachi, Pakistan; 6 Samira Imran, BScN. Indus Hospital and Health Network, Indus Hospital, Karachi, Pakistan; 7 Dr. Humza Thobani, MBBS. Section of Pediatric Surgery, Department of Surgery, Aga Khan University, Karachi, Pakistan; 8 Dr. Muhammad Aqil Soomro, MBBS, FCPS. Section of Pediatric Surgery, Department of Surgery, Aga Khan University, Karachi, Pakistan; 9 Dr. Saqib Qazi, MBBS, FCPS. Section of Pediatric Surgery, Department of Surgery, Aga Khan University, Karachi, Pakistan; 10 Dr. Saleem Islam, MD, MPH. Section of Pediatric Surgery, Department of Surgery, Aga Khan University, Karachi, Pakistan

**Keywords:** Tuberculosis, Pediatric abdominal tuberculosis, Surgical outcomes, Infectious diseases, Global surgery, Lower and middle-income country

## Abstract

**Objective:**

Abdominal tuberculosis (AbTB) is an extrapulmonary manifestation of tuberculosis (TB) that remains difficult to diagnose because its symptoms resemble many other gastrointestinal disorders. This study aimed to describe the clinical presentation, diagnostic evaluation, and treatment outcomes of pediatric AbTB treated at tertiary care centers in a lower middle-income country.

**Methodology:**

A retrospective review was conducted of children < 18 years who were diagnosed with AbTB between January 2012 and December 2024 at two tertiary care hospitals in Pakistan. Patients were categorized into medical and surgical groups based on definitive management. Descriptive and comparative analyses were performed. Statistical significance was defined as a p value ≤ 0.05

**Results:**

Ninety six children were included of which 26 were male. The mean age was 11.8 years, and the mean weight was 26 kg. Abdominal pain (82%) was the most common symptom followed by fever (59%) and vomiting (51%). The pattern of presenting symptoms did not differ significantly between the medical and surgical groups. Computed tomography (81%) was the most frequently used imaging modality. Standard antituberculosis therapy was provided to all patients. Surgical intervention was required in 41 children mainly for bowel obstruction or perforation. The mean length of stay was significantly longer in the surgical group (13 ± 9 days vs 8 ± 9 days, p=0.004). Overall, seven (7.3%) patients experienced mortality. Mortality was higher in the surgical group although this difference was not statistically significant (p=0.5).

**Conclusion:**

A large proportion of children present with advanced disease and require surgery. Improved access to imaging and strengthened referral and follow up systems are needed to achieve earlier diagnosis and reduce postoperative morbidity.

***Abbreviations:***

**TB:** Tuberculosis, **AbTB:** Abdominal tuberculosis,

**ATT:** Anti- tuberculous therapy, **LMIC:** Low- and middle-income countries.

## INTRODUCTION

Tuberculosis (TB) is a major contributor of national burden of disease in regions where socioeconomic barriers limit access to early diagnostic and therapeutic services. Over 10 million cases of TB were diagnosed worldwide in 2023 out of which more than 1.2 million were in children less than 14 years of age.^1^ While pulmonary disease accounts for the majority of pediatric TB cases, children have a disproportionately higher likelihood of extrapulmonary TB manifestations as compared to adults.^2^ Pakistan remains among the top five countries for high burden of TB and TB is a major contributor to mortality accounting for a mortality rate of 34 per 100,000.^3^ Almost 30% of all TB cases in the country present as extra pulmonary TB with more than 20% having abdominal involvement.^4^

Abdominal tuberculosis (AbTB) is a difficult entity to treat because of the vague and overlapping symptoms with other common gastrointestinal disorders.^5^ In pediatric patients it presents with combinations of abdominal discomfort, constitutional symptoms like fever, altered bowel habits and signs of acute abdomen, making the initial differential diagnosis broad and misleading.^6^ It is typically diagnosed using a combination of imaging, microbiologic testing and histopathologic assessment.^7^ The management of the disease varies between cases depending on disease severity at presentation. Although most patients respond well to standard anti-tuberculosis therapy (ATT), some require operative intervention for complications such as obstruction, perforation, or abscess formation.^8^

Understanding which clinical features and diagnostic patterns in children are associated with the surgical as compared to the medical management can help guide treatment plans in low- and middle-income countries (LMICs) like Pakistan. This study evaluated a large cohort of children diagnosed with AbTB at two high volume tertiary care centers, with emphasis on clinical presentation, diagnostic strategies, treatment modalities, and outcomes including the characteristics associated with surgical intervention.

## METHODOLOGY

We conducted a retrospective, multi-center study of all children less than 18 years old, diagnosed with AbTB at two high volume tertiary hospitals with dedicated pediatric surgical and infectious disease capabilities in Karachi, Pakistan. All eligible admissions between 1^st^ January 2012 through 31^st^ December 2024 were included in the study.

### Inclusion and Exclusion Criteria:

Cases were identified using hospital administrative and electronic medical record searches for relevant diagnostic and procedure codes (ICD-9 and ICD-10 codes) for abdominal and extrapulmonary TB. We included all children under 18 years with a diagnosis of abdominal tuberculosis established by one or more of the following; microbiologic confirmation of Mycobacterium tuberculosis from an intra-abdominal specimen or ascitic fluid, histopathologic evidence of granulomatous inflammation consistent with tuberculosis on tissue biopsy, or a combination of compatible clinical features and radiologic findings with a documented favorable response to a complete course of ATT as judged by the treating team. A favorable response to ATT was defined as partial or complete resolution of symptoms or documented microbiological clearance in patients with baseline microbiological positivity. Patients were excluded if essential medical records were missing or if they were transferred out before diagnostic workup could be completed.

The patients were classified into two primary management groups for analysis based on the treatment they received. Those treated with ATT without operative intervention were in the medical group while patients undergoing any operative intervention during management were placed in the surgical group. Extracted variables included demographics like the age, gender, and weight, the clinical presentation (symptoms and duration), past TB exposure or family history, baseline laboratory tests, microbiologic and histopathologic results, key findings on the imaging modalities, details of ATT, operative details (indication, procedure performed), and short-term outcomes (length of stay, complications, mortality). Length of hospital stay was defined as the interval from admission for the index illness to discharge. In hospital mortality referred to death during the same admission as the index diagnosis.

### Ethical Approval:

This study was reviewed by the Ethical Review Committee at our center and was deemed exempt from requiring ethical approval (ERC #2024- 10766- 31689) on December 2, 2024.

### Statistical analysis:

Data was exported into R version 4.5.1 for analysis. Normally distributed variables were reported as mean ± standard deviation, while non-normally distributed variables were reported as median with interquartile range (IQR). Categorical variables have been presented as numbers with their percentages. Categorical variables were compared using the Chi-square test or Fisher’s exact test as appropriate while the continuous data was compared using the Mann Whitney U test for non-parametric data. The statistical significance was defined as p value of less than 0.05.

## RESULTS

A total of 96 patients were diagnosed with AbTB over a 12 years period at two tertiary care centers. The mean age at presentation was 11.8 ± 3.8 years and the mean weight was 26 ± 11kg. 70 (73%) patients were female. 67 patients resided in urban areas of Sindh, and 15 patients were from rural districts. Four patients had permanent residence in Balochistan, and one patient was from Afghanistan.

**Fig.1:**
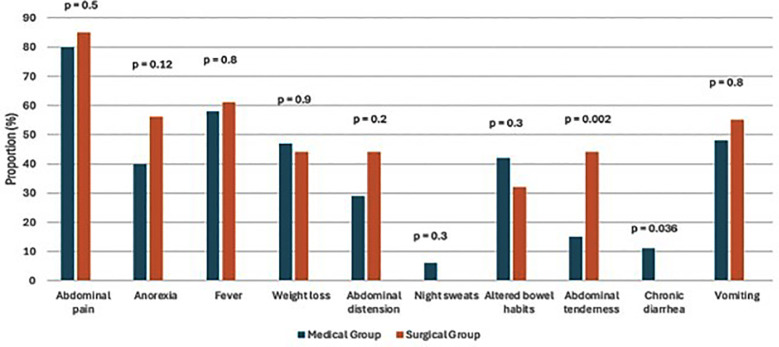
Clinical presentation of patients in surgical and medical group.

Abdominal pain was the most common presenting symptom followed by fever and vomiting. There were no significant differences in most presenting symptoms between the medical and surgical groups. Surgical patients were however more likely to have abdominal tenderness on examination (p= 0.002). The complete distribution of signs and symptoms for both groups is shown in Fig.1. The median duration of symptoms was 30 days with an interquartile range of 7 to 120 days. The wide interquartile range reflects substantial heterogeneity in symptom duration at presentation, with some patients experiencing prolonged symptoms prior to diagnosis. The duration of symptoms was shorter in the surgical group (20 days, IQR: 5 – 120) when compared to medical group (45 days, IQR: 7 – 90) however, this difference was not statistically significant (p=0.5). Previous therapy for TB had been received by 32 (35%) patients. Nineteen patients (21%) had a previous hospital admission due to AbTB. A family history or a confirmed contact was reported in 25 (27%) patients.

In the laboratory evaluation the mean hemoglobin was 10.15 ± 2.24 g/dL. 56 patients presented with anemia. The mean white blood cell count was 13 ± 8 x 10^9^/L. Erythrocyte sedimentation rate (ESR) was tested in 74 patients and the mean levels in this group were 33 ± 27. 36 (49%) patients had raised ESR. Acid fast bacillus (AFB) smear and culture were performed for 77 patients and two of these were positive. Gene expert testing was done for 50 patients, and the *Mycobacterium tuberculosis* was detected in 12 (24%) patients.

Computed tomography (CT) scan was performed in 78 patients (81%). The common abnormal findings are listed in Table-I. Abdominal ultrasound and plain X-ray was performed in 53 and 51 patients, respectively. On ultrasound nine patients had normal studies, 14 had lymphadenopathy, four had hepatomegaly, nine had dilated bowel loops and 18 had ascites.

**Table-I:** CT scan findings in patients with abdominal tuberculosis.

CT scan finding	n (%)
Findings suggestive of pulmonary involvement	22 (29)
Omental thickening and nodularity	20 (26)
Pneumoperitoneum	17 (22)
Bowel thickening	28 (36)
Mesenteric lymphadenopathy	50 (65)
Necrotic lymph nodes	28 (36)
Ascites	43 (56)
Bowel narrowing (stricturing)	5 (7)
Matted bowel	7 (9)

Ultrasound guided biopsy was available at one center, and it was performed in 12 patients for diagnostic confirmation. Exploratory laparotomy with biopsy was required in 28 patients to confirm the diagnosis. Microbiological confirmation was achieved for 13 patients and 27 had the diagnosis confirmed through histopathological evaluation. All patients received standard four drug ATT consisting of rifampin, isoniazid, ethambutol and pyrazinamide.

Surgical intervention was required in 41 patients. Of these 34 underwent bowel resection with or without stoma formation and seven patients had the peritoneal washout with drain placement. Stomas were created in 24 patients. The median duration of drain placement was 4.5 days (IQR: 3.75 to 7.25). Bowel obstruction in 29 patients and bowel perforation in 15 patients were the most common indications for surgery. Twelve patients with obstruction and two patients with perforation were not operated at the study centers. Three of these died before any intervention could be performed and eleven were lost to follow up. Among the patients who underwent surgery, 13 (32%) experienced a post operative complication (Table-II). Planned stoma reversal was performed in 12 patients at the study centers.

**Table-II:** Postoperative complication in patients that underwent surgery for the management of abdominal tuberculosis

Postoperative complications	n (%)
Surgical site infection	4 (10)
Sheath dehiscence	2 (5)
Small bowel obstruction	4 (10)
Abdominal collection	8 (20)
Sepsis	4 (10)
Enterocutaneous fistula	1 (2)
Anastomotic leak	0 (0)

The mean length of stay in those who got surgery was 13 ± 9 days which was significantly longer than those treated only with medical therapy (8 ± 9 days) (p=0.004). In hospital mortality occurred in three children (6%) managed medically and in four children (10%) who underwent surgery. This difference was not statistically significant (p=0.5). Only 25 patients were followed until completion of medical therapy, and four patients remain under follow up.

## DISCUSSION

Pediatric AbTB continues to present substantial diagnostic challenges in regions where TB is endemic. This multicentric study provides insight into disease patterns across two tertiary care centers in Pakistan and contributes to the limited contemporary literature from South Asia. The marked female predominance with a male to female ratio of one to 2.7 is consistent with reports from other developing countries where extra pulmonary tuberculosis appears more common in girls.^9^,^10^ The underlying reasons remain uncertain but may reflect biological susceptibility and social determinants that influence exposure and health seeking behavior.

The clinical manifestations of AbTB are difficult to interpret. Children frequently present with non-specific symptoms that resemble a wide range of gastrointestinal disorders. This overlap contributes to delays in diagnostic evaluation and subsequently delays initiation of treatment.^6^,^11^ Most patients in our cohort resided in urban and peri urban settings where access to timely diagnostic testing varies considerably. This likely explains the broad range in symptom duration which extended from a single day to almost twenty months. Moreover, the broad distribution of symptom duration likely reflects delayed presentation and diagnostic challenges associated with AbTB, particularly in settings where symptoms are nonspecific and access to timely diagnostic evaluation may be limited. Abdominal pain, fever, weight loss, and vomiting were the predominant presenting complaints which aligns with the classical symptom profile described in previous studies.^12^ The absence of significant differences in presenting symptoms between children who ultimately required surgery and those treated medically underscores the complexity of this disease. It indicates that early predictors of progression to complications remain difficult to identify in routine clinical practice. Although the duration of symptoms was shorter in the surgical group, this likely reflects delayed care seeking until the development of more severe clinical features. These findings highlight the need for heightened clinical vigilance in endemic regions and emphasize the importance of initiating ATT promptly when AbTB is suspected.

Microbiologic confirmation was uncommon in our cohort; a pattern that has been consistent in various studies on pediatric AbTB.^13^,^14^ AFB smears are often negative and add little diagnostic value in children with extra pulmonary disease. This limitation increases dependence on clinical assessment and imaging especially in settings where access to tissue diagnosis is restricted. Radiologic evaluation therefore becomes a central component of the diagnostic pathway. CT scan was performed in most patients and showed mesenteric lymphadenopathy and ascites as the common abnormalities. These findings are well recognized markers that help differentiate AbTB from disorders such as Crohn disease when histologic confirmation is not feasible.^15^ Ultrasound was performed in just over half of the cohort and frequently revealed nonspecific changes including hepatomegaly. A review by Vanhoenacker et al. has highlighted the value of CT in early recognition of features specific to AbTB.^16^ In high income countries CT is used more readily in children suspected of AbTB^17^ while in LMIC settings the cost of imaging often limits its access.^18^ Despite these constraints the timely use of CT should not be deferred as it guides both diagnosis and management. In our group, CT also detected pulmonary involvement in children without respiratory symptoms. This further emphasizes the importance of comprehensive imaging to optimize therapy and ensure appropriate follow up.

A high proportion of children in our study required operative intervention for bowel obstruction or perforation. Barot et al. have also reported obstruction and perforation as the most frequent indications for surgery in adolescents and adults with AbTB.^19^ The large number of surgical cases in our cohort likely reflects referral bias at tertiary centers where more complex cases tend to be concentrated. It may also reflect delays in presentation within the wider health system. Many children receive initial care at home or in primary care settings where symptoms such as abdominal pain and fever are often managed with simple medications and may not trigger timely referral. These delays allow the disease to progress until complications develop and surgery becomes unavoidable. This is the consequence of broader challenges in pediatric care in many LMICs where childhood illness is often managed initially at home or by non-specialists, and where symptoms in children may not prompt the same urgency of evaluation as in adults.^20^ These systemic factors contribute to delayed diagnosis and increase the risk that children present only after serious complications have developed.

Post operative morbidity was considerable, and most complications were due to infections. Intra-abdominal collection was the most common complication followed by sepsis and surgical site infections (SSI). These findings are consistent with reports from regional studies. Arbab et al. documented even higher rates of similar complications in children who required surgery for AbTB.^21^ Infectious morbidity continues to pose a significant challenge in many LMICs.^22^ Multiple factors likely contribute to this persistent burden. Antibiotic stewardship practices are often difficult to standardize in resource constrained environments. This inconsistency promotes the development of resistant organisms and reduces the effectiveness of empirical antibiotic regimens.^23^ Moreover, access to structured antibiotic guidelines varies across institutions. Adherence to infection control protocols is also challenging when clinical teams operate under heavy workload and limited infrastructure. In such circumstances the immediate focus is usually the provision of life saving surgical care. Preventive infection control measures therefore receive less emphasis in the daily workflow.

The consequences of post-operative infections extend well beyond the clinical episode. Recovery becomes prolonged and length of hospital stay increases. These additional days in the hospital escalate overall costs of care, placing substantial financial strain on families who frequently depend on out-of-pocket expenditure. Furthermore, prolonged admissions add to the workload of surgical services that already function with constrained capacity. This creates a cycle in which limited resources and high infection related morbidity continually reinforce each other.

### Limitations and Strengths:

There are several limitations to this study. The retrospective design restricts the ability to control for variability in clinical decision making and diagnostic evaluation. Documentation practices were inconsistent across records, which resulted in missing data for several important variables. Many patients received portions of their care at external hospitals, and their clinical information was not accessible, which made it difficult to determine the duration and completeness of therapy. Follow up was limited for a substantial proportion of the cohort because many families were unable to return for scheduled visits. These gaps reduce the precision of outcome assessment. Despite these limitations, the study has important strengths. It is multicentric and reflects practice across two major tertiary care centers. The sample size is considerable for a condition that is relatively uncommon in children. The comparison between medically treated and surgically treated patients provides insight into the spectrum of disease severity and the clinical factors associated with operative management.

## CONCLUSION

Strengthening structured clinical pathways for children with suspected abdominal tuberculosis is essential. Inequities in access to accurate diagnostic tools continue to delay recognition and timely referral. Standardized evaluation protocols that facilitate early imaging and coordinated multidisciplinary care may reduce progression to advanced disease. Limited follow up in this cohort reflects broader socioeconomic barriers that disrupt continuity of care and underscore the need for community-based support systems. Strengthening outpatient services and establishing reliable follow up pathways are critical to ensure treatment completion and minimize long term complications. The substantial surgical burden highlights the importance of early engagement of primary and secondary care providers. Future efforts should prioritize improving referral systems and developing pragmatic interventions that promote earlier diagnosis and reduce the need for operative management, thereby lowering morbidity and alleviating pressure on pediatric surgical services in TB endemic settings.

### Author contribution:

**JJ:** Data acquisition, statistical analysis, data interpretation, and manuscript drafting and is responsible for the integrity of the research.

**MOK**: Conception and design of the study and to manuscript writing.

**FZ:** Data acquisition and manuscript writing.

**RAF, MA,** and **SI:** Data acquisition across participating centers.

**HT:** The conception and design of the study.

**MAS and SQ:** study supervision.

**SI:** Data interpretation, critical revision of the final manuscript, and supervision.

All authors have reviewed and approved the final manuscript and agree to be accountable for all aspects of the work.
